# Factors Influencing Regular Exercise Habits of Women in Taiwan

**DOI:** 10.3390/ijerph182211960

**Published:** 2021-11-14

**Authors:** Chia-Che Liu, Liang-Ting Tsai

**Affiliations:** 1 Office of Physical Education and Sport Affairs, Feng Chia University, Taichung 407802, Taiwan; liucc@mail.fcu.edu.tw; 2 Institute of Education & Center of Teacher Education, Tzu Chi University, Hualien 97004, Taiwan

**Keywords:** exercise habit, Taiwan, female adults

## Abstract

This study employed Taiwanese women aged ≥18 years as the population and investigated their exercise habits, as well as the behavioral factors affecting such habits. In research on exercise, the relationship between exercise and sex is a crucial topic, and this relationship affects exercise promotion. Women’s exercise habits are influenced by traditional gender ideology, and women have long been excluded and treated unfairly in the realm of exercise. The study data were collected from a representative sample of 1113 Taiwanese women who participated in the 2011 Taiwan Social Change Survey (sixth phase, second wave). After removing missing values, the data of 1028 participants (46.39 ± 16.90 years) were analyzed. The results indicated that nearly half of the women had an exercise habit (48.3%). Those who were older, lived in a city (i.e., an area with a high level of urbanization), had a full-time job, had more favorable self-reported physical condition, and had enough income to meet their daily needs, were more likely to have an exercise habit.

## 1. Introduction

Improving quality of life and health are currently the focus of people’s attention. Krick and Sobal [1] confirmed that regular exercise positively affects physical and mental health. Exercise is beneficial for physical health, longevity, learning, and work performance. Due to social constraints, females in Taiwan may have less accessibility to regular physical activity. Lower rates of exercise may be due to personal factors (e.g., marital status, income, and health) and exercise environment implications; therefore, this study aimed to examine the factors influencing exercise behavior in this population.

Studies have shown that exercise is vital to the physical and mental states of humans [2,3]. Recreational exercise ensures physical fitness, relieves life pressures, improves work efficiency, cultivates group consciousness, boosts people’s teamwork spirit, corrects deviant behaviors, and increases the quality of life [3]. Godbey [2] reported that the benefits of recreational exercise differed among people with different perceptions and cognitive characteristics. Fu [4] and Strawbridge [5] noted that exercise not only promotes physical and mental health but also reduces stress. Moreover, exercise increases self-affirmation, facilitates positive cognition, furthers positive mental development, and prevents the occurrence of depression. From a physical perspective, the tangible benefits of exercise include the promotion of physical health and a reduction in the likelihood of mortality [4,5]. Wen et al. [6] revealed that, regardless of sex and presence of cardiovascular diseases, the mortality rate among people who engage in moderate physical activity (e.g., power walking) every day is 14% lower, and these people have, on average, three years longer life expectancy compared with people who do not exercise at all. Ratey and Sattelmair [7] also reported a lower incidence of cardiovascular diseases among people participating in recreational exercise.

In research on exercise, the relationship between exercise and gender has been a crucial topic affecting exercise promotion [3]. Women’s participation in exercise has been affected by gender definitions constructed on the basis of traditional gender ideology and logic, and women have long been excluded and unfairly treated in the exercise sphere. Shaw [8] claimed that women participate in exercise-related leisure activities less frequently than men and prefer social or cultural leisure activities. Although after marriage, women tend to have more free time than their husbands, women’s leisure activities are mainly family-centered because of family responsibilities. That is, women give less consideration to their needs and interests when choosing leisure activities than those of their family members. This is particularly true among married women who take care of children, and the housework is even more burdensome to professionally employed women [9,10].

Most people have realized that exercise improves their physical health, and women are encouraged to seek opportunities for exercising. Researchers have begun discussing the factors influencing women’s exercise habits. For example, Tsai et al. [3] contended that women are usually more dissatisfied with their physical appearance than men. In traditional gender logic, women are expected to be slim, sexy, and gentle. Women usually pay more attention to their physical appearance than men [11]. However, exercise has gradually developed into a manifestation of physical ability and fitness, and more women have begun paying attention to their physical health and fitness. Willis and Campbell [12] suggested that moderate exercise can reduce a person’s weight and body fat, and that increasing the intensity of exercise can rejuvenate physical appearance and further improve body image. Wilcox [13] reported that women who exercise regularly tend to have higher body image satisfaction. Loland [14] discovered that the body image of regular exercisers is more positive than that of non-exercisers, regardless of sex. Richman and Shaffer [15] found that participating in physical activities helps build a positive body image.

In addition to body image, women’s age, marital status, education level, employment status (i.e., whether or not she is a full-time employee), and degree of urbanization of the place of residence are all factors influencing women’s exercise habits and exercise engagement [3,4,16,17,18,19,20,21,22]. Wendel-Vosetal et al. [20] conducted a longitudinal analysis in the Netherlands and found that the length of time of recreational exercise was positively correlated with physical health in women. According to Pondè and Santana [21], engagement in recreational exercise is crucial for women who are dissatisfied with work or have low income because it helps them maintain their mental health. Thomsson [22] reported that Swedish women deem participation in recreational exercise essential to physical appearance and weight loss. Taiwanese women who are at the stage of just entering the workplace and marriage are responsible for caring for their family in addition to working. Therefore, they are left little time to participate in recreational exercise [3]. As women age, some gradually increase how often they exercise recreationally because of lower demands for family care or changes in their health.

In addition to personal factors, the exercise environment is another crucial factor affecting women’s exercise habits. Exercise venue and facilities are considered as the most basic elements for promoting exercise. An exercise habit can be developed through the improvement of knowledge, skills, and attitude; the exercise environment also has an essential effect [23]. However, gender differences in exercise habits caused by social structure and cultural power have resulted in the unfair distribution of exercise spaces in favor of men [24,25,26]. Therefore, gender differences exist in attitudes towards exercise and spatial perception. Compared with men, women are less satisfied with the experience of using a gym. Promoting exercise among women and encouraging them to develop exercise habits generally require more effort and attention than among men, but exercise space often serves as a factor that indirectly contributes to women developing exercise habits [27]. Therefore, understanding the exercise habits of women and which factors affect these habits is crucial to cultivating further exercise habits in this group. Numerous studies, such as Chuang [17], Li [28], Fu [4], and Tang [29], have explored the factors influencing women’s engagement in exercise, but the majority focused on a specific exercise or population. Comprehensive, nationwide research conducted in Taiwan remains lacking. With female adults in Taiwan as the study population, this study explored the status quo of the exercise habits of Taiwanese female adults, as well as the relevant behavioral factors influencing such habits.

## 2. Method

### 2.1. Participants

This study was a quantitative secondary analysis of data from the Taiwan Social Change Survey (TSCS), which was conducted on a sample of Taiwan’s population. The TSCS project was a cross-sectional study and conducted by the Institute of Sociology, Academia Sinica (although data gathered before the first wave of the third phase were obtained by the Institute of Ethnology, Academia Sinica), and sponsored by the National Science Council, Republic of China. The data employed in this research comprised the second-wave data of the sixth phase of the TSCS. The stratified three-stage probability proportional to size sampling was used to collect sample of 1113 Taiwanese women. After removing missing values, data from 1028 women (46.39 ± 16.90 years) were analyzed. The first national representative survey within the TSCS was completed in 1985. An annual TSCS consisting of two independent survey modules has been conducted since 1990. The main purpose of the series of surveys has been to obtain survey data through sampling various topics of social changes in Taiwan. All the data collected by the TSCS have been released to the academic community for research. The 2011 TSCS consisted of two modules. The second module, the health questionnaire, included the East Asian Social Survey 2010 and International Social Survey Program 2011 core questions, as well as items on other topics revealing the status of health and medical care in Taiwan [30].

### 2.2. Instruments

This study mainly explored the exercise habits of Taiwanese women aged ≥18 years, as well as the factors affecting such habits. The questionnaire for this study was designed through nine meetings of the expert panel. The psychometric characteristics of the questionnaire were also confirmed by pretesting [30]. The answer to the question “How often do you engage in exercises lasting at least 20 min that make you sweat more or breathe more quickly than usual?” from the 2011 TSCS (sixth phase, second wave) was used as the dependent variable; this question was rated using 1 = never, 2 = once a month or less (a few times a year or less), 3 = a few times a month, 4 = a few times a week, and 5 = every day. This study divided the participants into two groups (i.e., with and without an exercise habit) in accordance with their response. The women who responded “a few times a week” or “every day” were deemed as having an exercise habit, whereas the others were deemed as having no exercise habit. Regarding the independent variable, exercise habits may vary with demographic characteristics and socioeconomic status. After consulting the literature [3,4,16,17,18,19,20,21,22], this study took into consideration age, degree of urbanization of the place of residence, education level, marital status, employment status (i.e., whether or not the participant is a full-time employee), and body mass index (BMI). Because exercise environment is another crucial factor [23], this study included items related to outdoor environment in the questionnaire, such as “What is the air pollution level within a 1 km radius of your home?” and “Can you run or walk within a 1 km radius of your home?” Table 1 presents the coding of all the variables used.

### 2.3. Statistical Analysis

A descriptive statistical analysis was conducted to determine the demographics and characteristics of the survey sample. The chi-square difference test was performed to find the differences in characteristics between the participants and identify the factors that may affect the exercise habits of Taiwanese women. Multivariable logistic regression models were used to identify statistically significant associations of exercise habits with selected characteristics and factors. Logistic regression models were constructed and estimates of the strength of associations were obtained using the odds ratio (OR) and 95% confidence interval (CI). A *p*-value < 0.05 was considered statistically significant. To ensure the sample was representative of the entire population of Taiwanese women, data were analyzed using SPSS version 22.0 (SPSS Inc., Chicago, IL, USA) with appropriate sampling weights, which are included in the TSCS database.

## 3. Results

**Figure 1:**
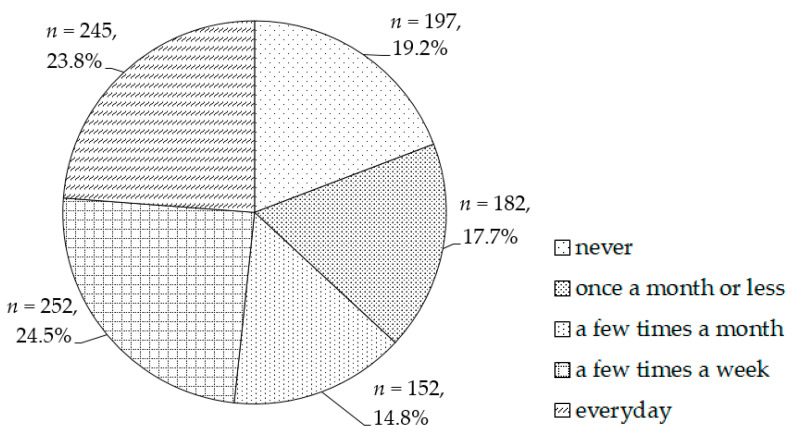
Distribution of the exercise habits.

The sample comprised 1028 Taiwanese women aged ≥18 years, nearly 20% of whom had never exercised. Among the participants, 24.5% and 23.8% engaged in exercise lasting at least 20 min and that made them sweat more or breathe more quickly than usual at least a few times a week and every day, respectively (Figure 1). Overall, 497 participants, accounting for 48.3% of the total sample, were determined to have an exercise habit, whereas the remaining 531 participants (51.7%) were not.

Nearly half of the participants (48.3%) had an exercise habit (Table 2). After the age of 30 years, an older participant was more likely to have an exercise habit. Compared with those living in areas with a low degree of urbanization, those residing in highly urbanized areas were more likely to have an exercise habit. A higher educational attainment was related to a lower likelihood of having an exercise habit. For example, among the participants who had an exercise habit, 38.8%, 23.8%, and 37.4% had an education level of junior high school or below, senior high school, and junior college or above, respectively, whereas, among those with no exercise habit, these percentages were 34.9%, 23.6%, and 41.5%, respectively. In total, 59.0% of the participants were married; 61.8% of the participants with an exercise habit were married. Regarding occupational status, 43.1% of the sample had a full-time job. Only 178 of the participants—accounting for only 17.4% of the total sample—had both a full-time job and an exercise habit. To determine whether the percentage of women with an exercise habit varied significantly with demographic characteristics (i.e., age, degree of urbanization of the place of residence, marital status, and occupational status), this study performed a chi-square contingency table test.

In total, 82.3% and 91.2% of the participants with and without exercise habits, respectively, claimed to be in fair and good physical condition (Table 3). Among the participants with an exercise habit, 66.8% stated that their income was enough to meet the needs of life. In terms of BMI, 33.8%, 60.4%, and 5.8% of the participants were overweight, had a normal or healthy weight, and were underweight, respectively. The percentage of participants with an exercise habit significantly varied among the subgroups with differing physical condition, income (i.e., their income met basic needs), and BMI, as demonstrated by the chi-square contingency table test.

Logistic regression analysis models were constructed to identify significant relationships between exercise habits and selected demographic characteristics and factors. The results are presented in Table 4. The factors associated with a greater prevalence of exercise habit for all participants were found to be (1) age 50–59 years (OR = 2.35, *p* = 0.003) or 60 years and older (OR = 2.63, *p* ≤ 0.001), (2) low degree of urbanization of residence (OR = 1.78, *p* = 0.003), (3) full-time employment (OR = 1.53, *p* = 0.006), (4) self-reported good health (OR = 2.29, *p* < 0.001), and (5) sufficient income to meet their daily needs (OR = 1.82, *p* < 0.001).

## 4. Discussion

Good exercise habits have been confirmed to considerably benefit a person’s mental and physical health. Motivations affecting exercise habits vary across individuals. Wendel-Vosetal et al. [20] conducted a longitudinal study on women in the Netherlands and discovered a positive correlation between the length of time of recreational exercise and physical health. Pondè and Santana [21] identified recreational exercise as a factor crucially affecting whether low-income female workers maintain favorable mental health. Warner-Smith and Brown [31] investigated women who worked as farmers in remote rural areas of Australia and discovered that their engagement in recreational exercise was greatly restricted because of factors such as a shortage of job opportunities, lack of public transportation, and unstable income. This result concurs with the finding of this study, in which Taiwanese female adults were the population, whereby Taiwanese women living in metropolitan areas and having enough income to meet their daily needs were found to be more likely to have an exercise habit than their suburban-living and lower-income counterparts, respectively. In Taiwan, job opportunities are more numerous, and salaries are generally higher in the cities, whereas job opportunities are fewer in remote suburban areas, resulting in a relatively unstable income. Therefore, the establishment of a regular exercise habit is more challenging for suburban residents than for urban dwellers.

Age was positively correlated with having an exercise habit. We discovered that the older participants (i.e., age ≥50 years) were more likely to have an exercise habit. The ORs for women aged 50–59 years and ≥60 years were 2.35 and 2.63, respectively. According to research, Taiwanese women who have just started working or recently been married may be responsible for caring for their family in addition to going to work, which leaves them little time to participate in recreational exercise. As women age, some begin to have more spare time to dedicate to exercise because of reduced family care responsibilities as their children grow up or due to early retirement. Therefore, we suggest that men should share the responsibility of family care in a timely manner to increase their wives’ leisure time in which they can develop an exercise habit. Otherwise, for young women who do not have the habit of exercise, due to the pressure of work and income considerations, it is really difficult to take the first step to exercise. However, moderate exercise can reduce stress and improve health. Therefore, it is recommended to experiment with different types of exercise (indoor/outdoor) and avoid engaging in a single sport, which can reduce the risk of exercise dependency and avoid the monotony of exercise or external weather factors that may affect the plan of continuous exercise. Exercising with pleasure and without psychological and physical burdens is an important factor for persistence. Therefore, it is recommended that young women start with light exercise, before slowly increasing the intensity of exercise to increase their sense of accomplishment and avoid losing the joy of exercise. Furthermore, the amount of exercise and activity in daily life can also be increased; for example, when taking public transportation, one can get off one stop earlier to increase walking time or one can take the stairs instead of escalators and elevators.

## 5. Conclusions

According to the results, women who were older, living in a highly urbanized area, with a full-time job, in good health, and with sufficient income to meet their daily needs were more likely to have an exercise habit.

In future studies, scholars should investigate differences between men and women in terms of exercise habits. In addition, students aged <18 years may face pressure to further their education, resulting in low exercise engagement. Factors affecting students’ exercise habits should also be explored. Only by being more inclusive can a more comprehensive in-depth understanding be gained of differences in exercise habits among Taiwanese people, as well as the factors that affect such habits at different stages of life.

## Figures and Tables

**Table 1:** Coding of all used variables.

Variable Name	Variable Description
Age	1 = 18–29; 2 = 30–39; 3 = 40–49; 4 = 50–59; 5 = ≥60
Degree of urbanization	1 = high (core urban region/general urban region); 2 = medium (emerging cities and townships/traditional industrial sector cities and townships); 3 = low (general townships and villages/rural areas)
Educational level	1 = junior high school or below; 2 = senior high school; 3 = junior college or above
Marital status	1 = married; 2 = others (single/divorced/widowed)
Occupational status	1 = full-time job; 2 = others (students/no job/part-time job)
What is the level of air pollution within a 1 km radius of your home?	1 = very serious; 2 = serious; 3 = not serious; 4 = not serious at all
Can you run or walk within a 1 km radius of your home?	1 = strongly disagree; 2 = disagree; 3 = neutral; 4 = agree; 5 = strongly agree
How do you feel about your health?	1 = bad; 2 = fair; 3 = good
BMI	1 = too light; 2 = standard range; 3 = too heavy
Is the current income enough to meet the needs of life?	1 = not enough; 2 = neutral; 3 = enough

**Table 2:** Participants’ demographic characteristics and survey responses (*n* = 1028).

Variables	Exercise Habits		Total	*p*-Value
	Yes (*n* = 497) *n* (%) = 48.3%	No (*n* = 531) *n* (%) = 51.7%		
Age group				<0.001 ***
18–29	83 (16.7)	125 (23.5)	208 (20.2)	
30–39	73 (14.7)	106 (20.0)	179 (17.4)	
40–49	97 (19.5)	114 (21.5)	211 (20.5)	
50–59	97 (19.5)	74 (13.9)	171 (16.6)	
≥60	147 (29.6)	112 (21.1)	259 (25.2)	
Degree of urbanization				0.031 *
High (core urban region/general urban region)	231 (46.5)	287 (54.0)	518 (50.4)	
Medium (emerging cities and townships/traditional industrial sector cities and townships)	175 (35.2)	171 (32.2)	346 (33.7)	
Low (general townships and villages/rural areas)	91 (13.7)	73 (13.7)	164 (16.0)	
Educational level ^a^				0.339
Junior high school or below	192 (38.8)	185 (34.9)	377 (36.8)	
Senior high school	118 (23.8)	125 (23.6)	243 (23.7)	
Junior college or above	185 (37.4)	220 (41.5)	405 (39.5)	
Marital status ^a^				0.045 *
Married	306 (61.8)	299 (56.4)	605 (59.0)	
Single/divorced/widowed	189 (38.2)	231 (43.6)	420 (41.0)	
Occupational status				<0.001 ***
Full-time job	178 (35.9)	265 (49.9)	443 (43.1)	
Others (students/no job/part-time job)	318 (64.1)	266 (50.1)	584 (56.9)	

* *p* < 0.05, *** *p* < 0.001; ^a^ number of missing data = 3.

**Table 3:** Distribution of factors that may affect the exercise habits.

Variables	Exercise Habits			*p*-Value
	Yes (*n* = 497) *n* (%) = 48.3%	No (*n* = 531) *n* (%) = 51.7%		
Air pollution				0.540
Very serious	31 (6.2)	23 (4.3)	54 (5.3)	
Serious	121 (24.3)	139 (26.3)	260 (25.3)	
Not serious	267 (53.7)	288 (54.2)	555 (54.0)	
Not serious at all	78 (15.7)	81 (15.3)	78 (15.7)	
Feel about your health				<0.001 ***
Bad	88 (17.7)	47 (8.8)	135 (13.1)	
Fair	203 (40.8)	232 (43.8)	203 (40.8)	
Good	206 (41.5)	251 (47.4)	457 (44.5)	
Income				<0.001 ***
Enough	203 (41.2)	158 (30.0)	361 (35.4)	
Neutral	126 (25.6)	157 (29.8)	283 (27.8)	
Not enough	164 (33.4)	211 (40.1)	375 (36.8)	
BMI				0.016 *
Too light	29 (5.8)	55 (10.4)	84 (8.2)	
Standard range	300 (60.4)	288 (54.2)	588 (57.2)	
Too heavy	168 (33.8)	188 (35.4)	356 (34.6)	

* *p* < 0.05, *** *p* < 0.001.

**Table 4:** Logistic regression of odds ratios (OR) of exercise among Taiwan women adults.

Variables	Odd	(95% CI)		*p*-Value	Cohen’s *f*^2^
Age group					0.009
18–29	1.00	-	-	-	
30–39	1.12	0.70	1.80	0.644	
40–49	1.50	0.91	2.47	0.114	
50–59	2.35	1.35	4.08	0.003 **	
≥60	2.63	1.53	4.54	<0.001 ***	
Degree of urbanization					0.003
Low	1.00	-	-	-	
Medium	1.33	0.99	1.79	0.055	
High	1.78	1.21	2.62	0.003 **	
Education level					0.001
Junior high school or below	1.00	-	-	-	
Senior high school	0.72	0.46	1.12	0.143	
Junior college or above	0.93	0.64	1.36	0.714	
Marital status					0.001
Single/divorced/widowed	1.00	-	-	-	
Married	1.03	0.74	1.42	0.881	
Air pollution					0.001
Very serious	1.00	-	-	-	
Serious	1.65	0.84	3.23	0.145	
Not serious	1.10	0.72	1.69	0.658	
Not serious at all	1.07	0.73	1.57	0.740	
Occupational status					0.006
Others (students/no job/part-time job)	1.00				
Full-time job	1.53	1.13	1.06	0.006 **	
Feel about your health					0.020
Bad	1.00	-	-	-	
Fair	1.07	0.80	1.42	0.653	
Good	2.29	1.49	3.52	<0.001 ***	
Income					0.013
Not enough	1.00	-	-	-	
Neutral	1.11	0.80	1.55	0.533	
Enough	1.82	1.31	2.52	<0.001 ***	
BMI					0.001
Too light	1.00	-	-	-	
Standard range	1.57	0.95	2.62	0.082	
Too heavy	1.16	0.67	2.01	0.587	
Cox and Snell *R^2^*	0.085				
Nagelkerke *R^2^*	0.113				

** *p* < 0.01, *** *p* < 0.001; OR = odds ratio; CI = confidence interval.

## Data Availability

All data can be downloaded freely from the following website: http://www.ios.sinica.edu.tw/sc/ (accessed on 20 October 2020).
